# Genetics of PlGF plasma levels highlights a role of its receptors and supports the link between angiogenesis and immunity

**DOI:** 10.1038/s41598-021-96256-0

**Published:** 2021-08-19

**Authors:** Daniela Ruggiero, Teresa Nutile, Stefania Nappo, Alfonsina Tirozzi, Celine Bellenguez, Anne-Louise Leutenegger, Marina Ciullo

**Affiliations:** 1 Institute of Genetics and Biophysics “A. Buzzati-Traverso”, National Research Council of Italy (CNR), Via Pietro Castellino, 111, 80131 Naples, Italy; 2 IRCCS Neuromed, Pozzilli, Isernia, Italy; 3 AORN Santobono-Pausilipon Hospital, Naples, Italy; 4 CHU Lille, U1167 - Labex DISTALZ - RID-AGE - Risk Factors and Molecular Determinants of Aging-Related Diseases, Inserm, Institut Pasteur de Lille, Univ. Lille, 59000 Lille, France; 5 UMR 946, Genetic Variation and Human Diseases, Inserm, 75010 Paris, France; 6 UMR946, Université Paris-Diderot, Sorbonne Paris Cité, 75010 Paris, France

**Keywords:** Genome-wide association studies, Quantitative trait

## Abstract

Placental growth factor (PlGF) is a member of the vascular endothelial growth factor family and is involved in bone marrow-derived cell activation, endothelial stimulation and pathological angiogenesis. High levels of PlGF have been observed in several pathological conditions especially in cancer, cardiovascular, autoimmune and inflammatory diseases. Little is known about the genetics of circulating PlGF levels. Indeed, although the heritability of circulating PlGF levels is around 40%, no studies have assessed the relation between PlGF plasma levels and genetic variants at a genome-wide level. In the current study, PlGF plasma levels were measured in a population-based sample of 2085 adult individuals from three isolated populations of South Italy. A GWAS was performed in a discovery cohort (N = 1600), followed by a de novo replication (N = 468) from the same populations. The meta-analysis of the discovery and replication samples revealed one signal significantly associated with PlGF circulating levels. This signal was mapped to the PlGF co-receptor coding gene *NRP1*, indicating its important role in modulating the PlGF plasma levels. Two additional signals, at the PlGF receptor coding gene *FLT1* and *RAPGEF5* gene, were identified at a suggestive level. Pathway and TWAS analyses highlighted genes known to be involved in angiogenesis and immune response, supporting the link between these processes and PlGF regulation. Overall, these data improve our understanding of the genetic variation underlying circulating PlGF levels. This in turn could lead to new preventive and therapeutic strategies for a wide variety of PlGF-related pathologies.

## Introduction

Placental growth factor (PlGF) is a member of the vascular endothelial growth factor (VEGF) family. It is a cytokine with a non essential role in healthy conditions, but with a specific involvement in several malignant, inflammatory and ischemic disorders^1–3^. High levels of circulating PlGF have been observed in individuals with various diseases such as cancer (breast^4^, melanoma^5^, leukemia^6^), immune diseases (rheumatoid arthritis^7^, Systemic Lupus Erythematosus^8^), metabolic syndrome^9^, coronary artery disease^10^ and neovascular age-related macular degeneration (nAMD)^11^. PlGF has recently emerged as a predictor of survival and cardiovascular risk and for cardiovascular risk stratification in patients with chronic kidney disease (CKD)^12^. Women affected by preeclampsia, a pregnancy-specific disorder characterized by the development of hypertension and proteinuria in the later stages of gestation^13^, show lower PlGF circulating levels compared to healthy pregnant women^14–16^. All these data show that the amount of PlGF has a relevant impact on the determination of pathological conditions. Moreover, since PlGF levels can be pharmacologically modifiable, understanding the determinants of circulating PlGF could support efforts directed at risk prediction, prevention and therapy.

Although the heritability of circulating PlGF levels is around 40%^17^, the knowledge about the genetic factors modulating circulating PlGF levels is limited. Indeed, no studies have assessed the relation between circulating PlGF levels and genetic variants at the genome-wide level. In the present work, we have conducted the first genome-wide association study (GWAS) for PlGF in a general population sample with a deep genomic coverage based on imputation to the 1000 Genomes panel, to identify genetic variants that explain variation in circulating PlGF concentrations.

## Results

### GWAS of PlGF plasma levels: discovery, replication and meta-analysis

A GWAS meta-analysis of PlGF circulating levels was performed in 2068 individuals from three isolated populations of Cilento, South Italy^18,19^. Characteristics of the study participants are presented in Table 1. A sample of 1600 individuals was used as discovery cohort. An additional sample of 468 individuals from the same populations was used in the de novo replication stage. The replication sample was younger than the discovery sample, no other differences between the two cohorts were observed.

**Table 1:** Characteristics of study participants from the discovery and replication cohorts.

Cohort	Discovery	Replication
No of Individuals	1600	468
Women (%)	55.5	54.1
Age (mean ± SD)	52.5 ± 19.7	35.2 ± 25.2
PlGF (pg/ml)		
All		
Median	12.5	12.2
Range	4.8—51.2	5.6—69.3
Men		
Median	13.2	13.0
Range	5.6—34.2	5.6—57.7
Women		
Median	12.0	11.7
Range	4.8—51.2	5.9—69.3

**Figure 1:**
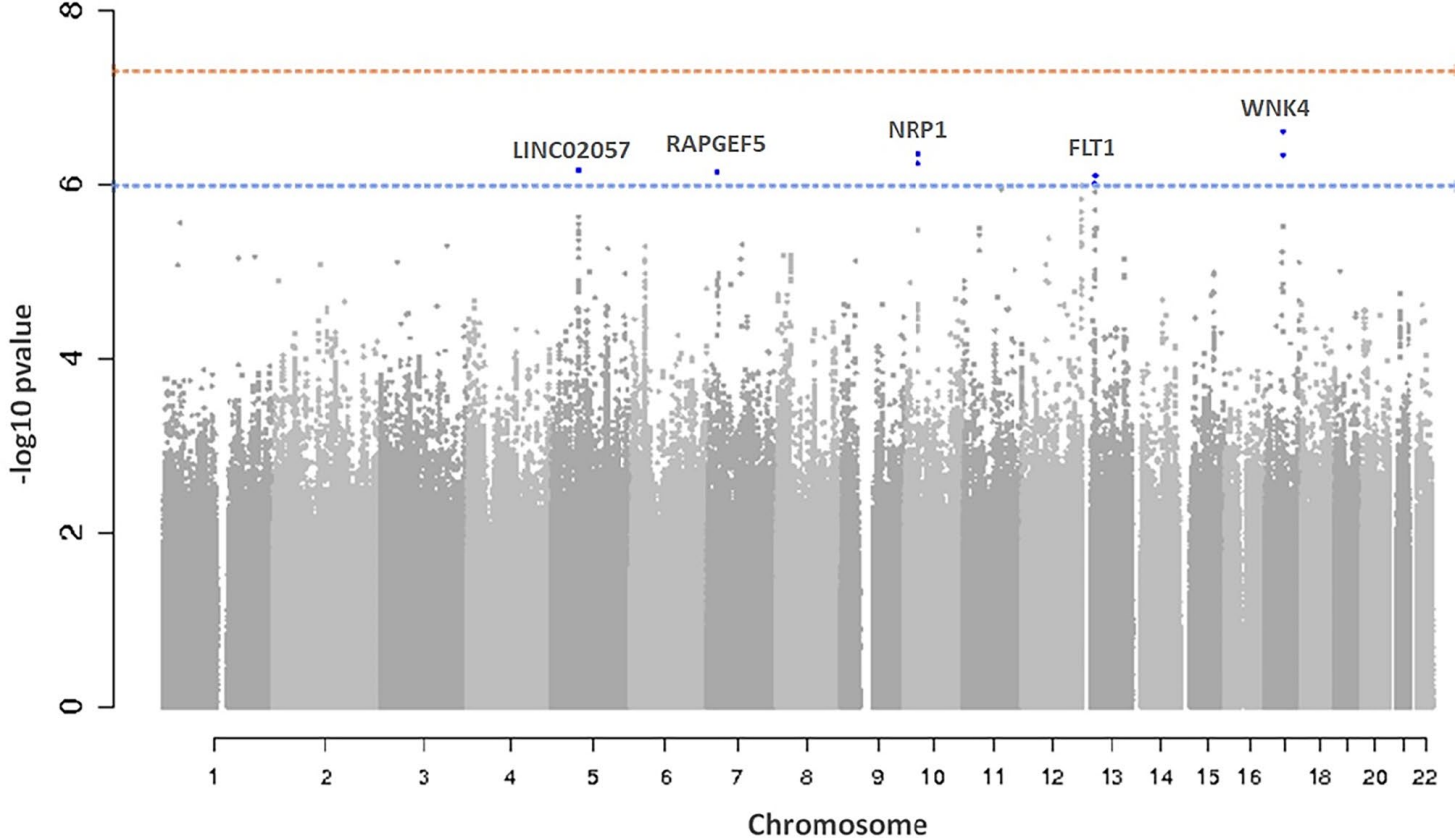
Manhattan plot of genome-wide association results in discovery analysis. Manhattan Plot showing − log10(*p*-values) for all SNPs of the PlGF discovery GWAS ordered by their chromosomal position. The blue dashed line indicates the suggestive association threshold (*p*-value < 1 × 10^–6^), while the red dashed line indicates the genome-wide significant threshold (*p*-value < 5 × 10^–8^). Blue dots are the SNPs associated at the suggestive significance level (*p*-value < 1 × 10^–6^). For each locus, the nearest gene is reported.

In the discovery GWAS 8,281,256 autosomal SNPs were investigated for association with PlGF plasma levels. A Manhattan plot and a Q-Q plot of the association results are reported in Fig. 1 and S1 Figure, respectively. Although none of the signals in the discovery cohort reached genome-wide significance (*p*-value < 5 × 10^–8^), 88 variants were associated at *p*-value < 1 × 10^–5^ and, among those, 7 variants were associated at *p*-value < 1 × 10^–6^. A linkage disequilibrium (LD)-based clumping procedure revealed 5 independent signals, suggestively associated with PlGF plasma concentration at a *p*-value < 1 × 10^–6^. The regional association plots reported in the S2 Figure provide a detailed overview of those loci. Those variants were carried forward to a de novo replication. Summary results for main associations for PlGF plasma levels are shown in Table 2. Overall, in the meta-analysis of discovery and replication, a variant on chromosome 10p11.22 reached a genome-wide significant *p*-value; two additional signals, on chromosomes 13q12.3 and 7p15.3, although didn’t reach the genome-wide significance, showed an effect in the same direction between discovery and replication and a lower *p*-value in the meta-analysis compared to that of the discovery stage. Therefore, based on these criteria, we considered the 13q12.3 and 7p15.3 loci as replicated signals. In the locus on chromosome 10p11.22, the most significantly associated variant was rs17296631 (*p*-value = 8.36 × 10^–9^) located upstream Neuropilin 1 (*NRP1*) gene, which encodes for a co-receptor of PlGF protein. The rs9551465 variant (*p*-value = 7.84 × 10^–8^), identified on chromosome 13q12.3, was located in the 3’UTR of the Fms Related Tyrosine Kinase 1 (*FLT1*) gene, coding for the PlGF receptor Flt-1/VEGFR-1. The variant rs77619310 (*p*-value = 5.27 × 10^–7^) on chromosome 7p15.3 was located in an intron of the *RAPGEF5* gene.

**Table 2:** Top variant results from discovery and replication meta-analysis.

Variant rsID	Chr	Position (hg19)	Nearest Gene	Position respect to the Nearest Gene	EA	EAF	Discovery			Replication			Meta-analysis			
							Beta	SE	*p*-value	Beta	SE	*p*-value	Effect direction	Beta	SE	*p*-value
rs10037643	5	60,565,864	*LINC02057*	5.1 Kb upstream	A	0.46	0.023	0.005	6.92 × 10^–7^	0.002	0.009	0.822	+ +	0.018	0.004	7.00 × 10^–6^
rs77619310	7	22,252,636	*RAPGEF5*	Intronic	A	0.04	0.058	0.012	7.22 × 10^–7^	0.031	0.025	0.219	+ +	0.053	0.011	5.27 × 10^–7^
rs17296631	10	33,766,736	*NRP1*	142.9 Kb upstream	T	0.04	0.064	0.013	4.47 × 10^–7^	0.066	0.024	5.47 × 10^–3^	+ +	0.065	0.011	8.36 × 10^–9^
rs9551465	13	28,962,757	*FLT1*	Intronic	T	0.54	0.023	0.005	9.93 × 10^–7^	0.019	0.008	0.024	+ +	0.022	0.004	7.84 × 10^–8^
rs61755606	17	40,940,579	*WNK4*	Intronic	A	0.11	0.039	0.007	2.50 × 10^–7^	0.013	0.016	0.399	+ +	0.034	0.007	5.25 × 10^–7^

*Chr* chromosome, *EA* effect allele, *EAF* effect allele frequency, *SE* standard error, Effect Direction: direction of the effect allele on PlGF levels in Discovery and Replication.

The three most associated loci collectively explained 3.68% of the PlGF phenotypic variance.

### Colocalization analysis

To verify if the variants associated with PlGF circulating levels directly act through gene expression regulation in a particular tissue, and considering that, given the involvement of PlGF in different pathological conditions, it might exert a role in several tissues, we tested the three signals on chromosomes 7, 10 and 13 for colocalization with expression quantitative trait loci (eQTL) in all the 48 tissues from Genotype-Tissue Expression (GTEx)^20^ using the *coloc* program^21^ (https://rdrr.io/cran/coloc/man/coloc.abf.html).

We highlighted a very high posterior probability of colocalization (H4 = 0.96) in the Artery Tibial tissue for the rs77619310 variant, which is the best GWAS hit on chromosome 7, and an eQTL for the *RAPGEF5* gene. The A allele, reported to determine an increase of *RAPGEF5* mRNA levels, was also associated with higher levels of PlGF.

This analysis also revealed that the rs9551465, the most associated variant on chromosome 13, showed the best colocalization signal (H4 = 0.79) in the Thyroid tissue, where it is also reported as an eQTL for the *FLT1* gene. In this case the allele associated with an increase of PlGF levels was correlated with a lower expression of the *FLT1* gene.

The best signal on chromosome 10, the rs17296631, is in complete LD with rs145141871, the second most associated variant in this locus, and both are reported as eQTLs for the *NRP1* gene in the Brain Anterior cingulate cortex tissue. For both variants, the alleles that lead to higher expression levels of the *NRP1* gene are also associated with an increase of PlGF levels. Despite this evidence, *coloc* did not reveal significant colocalization evidence, confirming that in such a situation of complete LD between two variants, the program shows uncertainty in identifying the causal one^21^.

### Transcription-wide and gene-based association analyses

To further identify loci associated with PlGF levels, we performed a transcription-wide association analysis (TWAS) and a gene-based analysis.

In our study, TWAS analysis identified several genes in different genomic regions whose expression in particular tissues, is associated with circulating levels of PlGF protein. The list of the 52 gene/tissue pairs, showing a significant association at FDR (< 0.05), is reported in the S1 Table. Interestingly, the majority of them are located on chromosome 6p22.1-p21.33, in the HLA region. These results suggest that multiple variants, each likely with a marginal level of significance and located in the HLA genomic region, might contribute, in aggregate, to the regulation of the PlGF circulating levels.

The top 10 genes from the VEGAS2 gene-based analysis^22^ are reported in the S2 Table. Although none of the analysed genes showed a statistically significant False Discovery Rate (FDR)-adjusted *p*-value, the best association was found for the *FLT1* gene, in accordance with the result obtained in the GWAS. Furthermore, except for the *SMIM15-AS1* gene on chromosome 5, the other associated genes are all located in the HLA region on chromosome 6, in conformity with the results obtained in the TWAS analysis, and again suggesting an involvement of this locus on the regulation of the PlGF levels.

### Pathway analysis

To discover biological pathways involved in the modulation of the circulating levels of PlGF, we performed an enrichment analysis using the GSA-SNP2 program^23^. The analysis highlighted 27 Gene Ontology (GO) significantly enriched pathways (q-value < 0.05) (16 Biological Processes, 5 Cellular Components and 6 Molecular Functions), reported in Table 3. In line with the results obtained in the TWAS and the gene-based association analysis, the majority of the significantly enriched pathways were related to immune response. In fact, the GO terms summarization analysis performed by REVIGO program^24^ evidenced that 15 out of the 16 Biological Processes were linked to the immunoglobulin mediated immune response, and that 2 Cellular Components and one Molecular Function were related to the MHC class II complex. Also, 3 GO terms were linked to the lumenal side of the endoplasmic reticulum membrane. Other significantly over-represented pathways were the N-glycan processing, the oxidoreductase activity, the mismatched DNA binding, the folic acid binding and the AU-rich element binding.

**Table 3:** Significantly enriched pathways.

Gene ontologies	GO term	q-value	Dispensability
BP	GO:0006491N-glycan processing	0.042	0
BP	GO:0016064 immunoglobulin mediated immune response	7.23 × 10^–4^	0
*BP*	*GO:0006959 humoral immune response*	*0.036*	*0.692*
*BP*	*GO:0042129 regulation of T cell proliferation*	*0.035*	*0.637*
*BP*	*GO:0002377 immunoglobulin production*	*3.69* × *10*^*–3*^	*0.632*
*BP*	*GO:0002889 regulation of immunoglobulin mediated immune response*	*0.040*	*0.947*
*BP*	*GO:0002381 immunoglobulin production involved in immunoglobulin mediated immune response*	*0.035*	*0.952*
*BP*	*GO:0034112 positive regulation of homotypic cell–cell adhesion*	*0.036*	*0.722*
*BP*	*GO:0019724 B cell mediated immunity*	*1.42* × *10*^*–3*^	*0.924*
*BP*	*GO:0002712 regulation of B cell mediated immunity*	*0.040*	*0.947*
*BP*	*GO:0002440 production of molecular mediator of immune response*	*5.25* × *10*^*–3*^	*0.627*
*BP*	*GO:0002460 adaptive immune response based on somatic recombination of immune receptors built from immunoglobulin superfamily domains*	*0.043*	*0.933*
*BP*	*GO:0002637 regulation of immunoglobulin production*	*0.049*	*0.888*
*BP*	*GO:1903039 positive regulation of leukocyte cell–cell adhesion*	*0.040*	*0.979*
*BP*	*GO:0002455 humoral immune response mediated by circulating immunoglobulin*	*0.035*	*0.952*
*BP*	*GO:0050870 positive regulation of T cell activation*	*0.039*	*0.933*
CC	GO:0042613 MHC class II protein complex	1.83 × 10^–5^	0
*CC*	*GO:0042611 MHC protein complex*	*3.21* × *10*^*–3*^	*0.501*
CC	GO:0071556 integral component of lumenal side of endoplasmic reticulum membrane	4.37 × 10^–3^	0.032
*CC*	*GO:0098576 lumenal side of membrane*	*0.035*	*0.513*
*CC*	*GO:0098553 lumenal side of endoplasmic reticulum membrane*	*4.37* × *10*^*3*^	*0.606*
MF	GO:0016641 oxidoreductase activity, acting on the CH-NH2 group of donors, oxygen as acceptor	6.87 × 10^–3^	0
MF	GO:0030983 mismatched DNA binding	1.42 × 10^–3^	0
MF	GO:0032395 MHC class II receptor activity	8.59 × 10^–5^	0
MF	GO:0005542 folic acid binding	0.036	0.066
MF	GO:0017091 AU-rich element binding	0.040	0.149
MF	GO:0016638 oxidoreductase activity, acting on the CH-NH2 group of donors	0.035	0.299

Gene Ontologies: Molecular Function (MF), Biological Process (BP), Cellular Component (CC). Dispensability represents a measure of the non-redundancy of a GO term with respect to other semantically close terms. Representative, non-redundant GO terms are given in black, the other cluster members are in italics.

## Discussion

Our study is the first GWAS of circulating PlGF levels. It was undertaken in 2068 individuals from three population isolates of the Cilento area, South Italy, and represents the largest survey of PlGF measurement in a population-based sample. In this study, we have identified 3 chromosomal regions (7p15.3, 10p11.22 and 13q12.3) harboring variants associated with PlGF plasma levels. The three top variants in these loci explain about 4% of PlGF phenotypic variance.

The newly identified regions include many interesting and plausible candidate genes.

The lead variant on chromosome 10, rs17296631, is located in an intergenic region upstream the *NRP1* gene. This gene encodes for the Neuropilin-1, a membrane protein devoid of tyrosine kinase activity which acts as a co-receptor for PlGF. Recent studies suggest that NRP-1, through the binding of PlGF and other growth factors, can play an important role in the activation of specific signal transduction pathways also independently of other membrane receptors^5,25^. A positive correlation of expression of PlGF and NRP-1 has been observed in breast cancer^4^, and PlGF activation of NRP-1 could also promote tumor cell survival in a paracrine manner in a mouse model of medulloblastoma^25^. These observations are in line with the results of the eQTL analysis, in which the T allele of the rs17296631 variant is associated with an increase of both PlGF levels and *NRP1* gene expression.

On chromosome 13, the rs9551465 is located in an intron of the *FLT1* gene. This gene encodes for a tyrosine-protein kinase, Flt-1, belonging to the vascular endothelial growth factor receptor (VEGFR) family, which functions as a receptor for VEGFA, VEGFB and PlGF. The protein is also present in a soluble form, sFlt-1, lacking of the transmembrane and intracellular domains. The PlGF binding to Flt-1 stimulates angiogenesis via both direct and indirect mechanisms: the activation of Flt-1 by PlGF results in phosphorylation of specific tyrosine residues in Flt-1 and downstream signaling different from those activated by VEGFA binding; also, it has been proposed, based on in vitro data and overexpression studies, that the binding of PlGF to Flt-1 induces pro-angiogenic effects as PlGF shifts VEGFA from Flt-1 to VEGFR2, enhancing the effects of VEGFA^26^. In the present study, we have found that the PlGF associated rs9551465 variant in the *FLT1* gene is also an eQTL for *FLT1*: in particular, the variant allele (T) is associated with higher PlGF protein levels and with a lower *FLT1* gene expression. This is consistent with the observation that a higher expression of Flt-1, acting as a “decoy” for PlGF, can determine lower detectable circulating levels of the ligand protein^14^.

The third associated variant, rs77619310, is located on chromosome 7p15.3 in an intron of the *RAPGEF5* gene. This gene, encoding for a guanine nucleotide exchange factor (GEF) for the GTPases Rap1, Rap2 and M-RAS, serves as RAS activator by promoting the acquisition of GTP to maintain the active GTP-bound state and is a key link between cell surface receptors and RAS activation^27,28^. Interestingly, Rap1, activated by different GEFs, including RAPGEF5, acts as a regulator of several basic cellular functions such as adhesion, polarity, differentiation and growth^29,30^. In the endothelium, Rap1 is a key positive regulator of angiogenic process^31^. It functions downstream of the main angiogenic growth-controlling receptors in endothelial cells, including VEGFA^32^. Also, Rap1 promotes VEGF-mediated angiogenesis through the activation of VEGFR2^33^ and is capable of regulating endothelial barrier permeability by VEGF stimulation^34^.

In a previous candidate gene study, we reported the association of the *PlGF* gene locus with the levels of the protein in plasma^17^. More recently, another study has found an association between an additional polymorphism in the *PlGF* gene and the protein plasma levels^35^. In the current study, no variants in the *PlGF* gene region reached the statistical significance, however, some SNPs located between 5 and 20 kb upstream of the gene were nominally associated with PlGF levels (rs4903273, *p*-value = 2.04 × 10^–3^) and showed a moderate LD (*r*^2^ = 0.56) with the rs2268614 SNP described in our previous work^17^.

An implication of HLA genes in the regulation of PlGF levels was detected at statistical significance in the TWAS and also suggested by a gene-based association analysis, confirming an increased power of TWAS analysis which takes advantage from gene expression level data^36^. In particular, such genes belong to the HLA class III and are involved in both the immune system function and the angiogenic process. In detail, gene expression levels of *LY6G5B* and *LY6G5C*, belonging to the cluster of leukocyte antigen-6 (*LY6*) genes, showed an association with PlGF circulating levels in multiple tissues. Little is known about the proteins coded by these two genes, however other members of the LY6 protein family, expressed in various types of tissues and at different stages of cell differentiation, are involved in cell proliferation, cell migration, cell–cell interactions, immune cell maturation, macrophage activation, and cytokine production^37,38^, and their overexpression or dysregulation is associated with tumorigenesis and autoimmune diseases^39^. Also, the *C4A* gene, encoding for the acidic form of the complement factor 4, plays a pivotal role in the activation of immune defenses and the clearance of immune complexes or apoptotic debris in vitro and in vivo studies^40,41^. A lower number of gene copies of *C4A* has been linked to an increased risk for different autoimmune diseases, such as Systemic Lupus Erythematosus, Type 1 Diabetes and Juvenile Dermatomyositis^42–44^. In addition, the deficiency of the *C4A* gene has also been associated with preeclampsia, a well-established PlGF related disease^14–16,45,46^, with a lower gene copy number associated with an increase of disease severity, supporting the importance of the classical pathway of the complement system in this pathology^47^. Also, higher levels of C4A have been observed in patients with neovascular age-related macular degeneration^48,49^, an ocular pathology also characterized by an increase of PlGF levels^11^. Finally, some evidences link the pseudogene *MICD* to the immune system: in fact, SNPs in this gene region have been associated with eosinophil, basophil and granulocyte count^50^ and with different autoimmune disease, such as vitiligo^51,52^, Graves' disease^53^ and psoriatic arthritis^54^.

Other genes in the same region, the Dimethylarginine dimethylaminohydrolase-2 (*DDAH2*) and the *TCF19,* are implicated in the angiogenic process. The *DDAH2* gene encodes for an enzyme that positively regulates the nitric oxide (NO) generation by metabolizing the asymmetric dimethylarginines (ADMA), which are inhibitors of the nitric oxide synthase (NOS) activity^55^. DDAH2 acts as a key regulator of the angiogenesis: in fact, its overexpression enhances the proliferation and migration of endothelial cells through the induction of expression and secretion of VEGF, both regulating the production of endothelial NO through the stimulation of endothelial NOS (eNOS) activity^56^, and in an eNOS-independent manner, via the activation of Sp1 binding site of the VEGF promoter^57^. DDAH2 is considered an antiatherosclerotic molecule: in fact, hypermethylation of *DDAH2* promoter, accompanied by its reduced expression, correlates with endothelial dysfunction in patients affected by Coronary Artery Disease^58^. The inhibition of *DDAH2* has also been correlated with an attenuation of aberrant angiogenesis and with the improvement of vascular regeneration in mice models of oxygen-induced retinopathy (OIR)^59^, and similar effects on the retinal vasculature have also been observed in PlGF deficient OIR mouse models^1^. Also, decreased expression levels of *DDAH2* have been observed in women affected by preeclampsia^60,61^.

The *TCF19* gene encodes a transcription factor containing a PHD-type zinc finger domain and plays a role in the proliferation and apoptosis of pancreatic beta cells^62^. SNPs in this gene have been associated with chronic Hepatitis B, Type 1 and Type 2 Diabetes^63–65^. Recently its overexpression has been linked to an increase of cell proliferation in different types of cancer^66,67^.

It is of interest to note that, although colocalization and TWAS analyses both use GTEx expression data, the PlGF associated genes, which colocalize with eQTL signals, were not detected by TWAS analysis. From this observation, we noted that in the PredictDB database (http://predictdb.org/) the prediction models used for the imputation of the expression levels are present, depending on the tissue, for a limited and variable number of genes. Also, imputation is performed using a less dense panel of variants compared to that used in the GWAS. Both these circumstances might explain the possibility of bypassing some association signals in the TWAS. In our analysis, *NRP1* and *FLT1* imputed genes expression levels are missing for those tissues (Brain Anterior cingulate cortex and Thyroid, respectively) in which the colocalization was identified. For the *RAPGEF5* gene, for which a very high posterior probability of colocalization was evidenced in the Artery Tibial tissue, the TWAS failed to detect an association (*p*-value = 0.017), probably because imputed expression levels were obtained using a reduced number of variants (N = 27), not including any of the PlGF associated variants detected in the GWAS at this locus.

The pathway analysis also revealed an over-representation of several pathways linked to the immune system. To date, only a few studies have linked PlGF to the adaptive immune response. Lin et al. reported the evidence of immunosuppressive properties of PlGF. They found that PlGF inhibited the activation and maturation of human dendritic cells, differentiated from CD14 + monocytes, through the NF-κB signaling pathway. PlGF-treated dendritic cells resulted in the downregulation of maturation markers CD80, CD86, CD83, CD40, and MHC-II expression as well as the inhibition of IL-12, IL-8, and TNF-α production in response to Lipopolysaccharide stimulation, in respect to untreated dendritic cells. In addition, treatment of dendritic cells with PlGF resulted in the suppression of naïve CD4^+^ T cell proliferation in an allogeneic mixed lymphocyte reaction. The results from this study indicate that PlGF can downregulate type 1 T helper immune responses by modulating the function of dendritic cells^68^.

Han et al. reported the effect of PlGF on the regulatory B cells (Bregs), a subset of B lymphocytes with a pivotal role in regulating immune responses involved in inflammation, autoimmunity and cancer. Using surgically removed glioma tissues, they demonstrated that glioma cells release exosomes carrying PlGF. When purified naïve B cells captured the PlGF-containing exosomes from glioma cells, they differentiated into TGF-β^+^ Bregs able to suppress the CD8^+^ T cell activities. Further, the treatment of glioma cells with an anti-PlGF antibody (TB-403), a (PlGF)-specific inhibitor, completely suppressed the expression of the TGF-β, while the exposure of glioma cells to PlGF, upregulated the expression of the TGF-β^69^.

A recent work has demonstrated that PlGF is selectively secreted by the helper T cells (T_H_17), a subset of inflammatory T cells that, producing IL-17, contribute to autoimmunity and tissue damage^70^ and which dysregulation is associated with various autoimmune diseases, including multiple sclerosis and rheumatoid arthritis^71,72^. In the same article, the authors demonstrate that T cell-produced PlGF is functionally active in promoting angiogenesis and that PlGF stimulates T_H_17 cell differentiation by activating STAT3 via binding to the Flt1 and NRP1 receptors. Also, they show that the overexpression of PlGF in T cells exacerbates disease in mice with collagen-induced arthritis and that PlGF concentrations also correlated with IL-17 concentrations in synovial fluid from patients with rheumatoid arthritis. Overall, they provide an insight into the links between angiogenesis, T_H_17 cell development, inflammation and autoimmunity, emphasizing the importance of PlGF in these processes^70^. Previously, Kang et al., using a PlGF-overexpressing transgenic mice model, showed that PlGF secretion was upregulated in isolated T-cells, suggesting PlGF as a regulator of T-cell differentiation. In addition, the authors evidenced that the CD4 + T-cells isolated from the spleen of transgenic mice indicated greater inflammatory T_H_1 and T_H_17 helper T-cell differentiation, thereby emphasizing the role of PlGF in T-cell differentiation and development^73^.

In conclusion, in this study we have identified some genes and pathways, known to be implicated in the angiogenesis process and the immune response, that are associated with the variation of circulating PlGF. The identification of those genes corroborates the link between PlGF protein levels and immune system function and could lead to new preventive and therapeutic strategies in immune and/or angiogenesis-related diseases in which PlGF has been implicated.

## Methods

### Population samples and PlGF measurement

The discovery sample includes 1600 volunteer individuals recruited through a population-based sampling strategy in three small isolated villages of the Cilento region, South Italy (Campora, Gioi and Cardile). A de novo replication was performed in additional 468 subjects from the same villages^18,19^. A subset of this sample (N = 871) was used in our previous study on the genetics of PlGF plasma levels^17^.

The Cilento study was approved by the ethics committee of “Azienda Sanitaria Locale Napoli 1” (Medical committee, in 2003 protocol #291 and #113, and in 2007 protocol #556) and the ethics committee “Carlo Romano” University of Naples “Federico II” (Research committee, in 2013 protocol #231/13). The study was conducted according to the criteria set by the declaration of Helsinki and each subject signed an informed consent before participating to the study.

Blood samples were collected in the morning after the participants had been fasting for at least 12 h. Aliquots of plasma were immediately prepared and stored at − 80 °C and were subsequently used for the assessment of PlGF levels. PlGF levels (pg/ml) were measured using an electrochemiluminescence immunoassay on the Elecsys2010 analyzer (Roche Diagnostics, Mannheim, Germany).

Pregnant women were excluded from the study because of their high level of PlGF in the plasma^17^.

A logarithmic transformation was applied to the PlGF levels to normalize the trait distribution and the transformed trait was used in all subsequent statistical analyses.

The Mann–Whitney U test was used to compare median PlGF plasma levels among the samples.

### GWAS and replication study

Genotyping in the discovery sample was performed with 370 K and OmniExpress Illumina chips, phasing and imputation were conducted separately by platform with the MaCH^74^ (http://csg.sph.umich.edu/abecasis/mach/index.html) and minimac^75^ (https://genome.sph.umich.edu/wiki/Minimac) software respectively, using 1000 Genomes Phase 1 v3 data as reference. Quality control filters applied before imputation were call rate > 95% for SNPs and samples and minor allele frequency (MAF) > 0.01. GWAS was carried out through a mixed model linear regression where the variance/covariance matrix is the genomic kinship to account for relatedness between individuals. Age and gender were used as covariates and an additive genetic model was considered. The analysis was performed with GenABEL R package^76,77^ (https://cran.r-project.org/src/contrib/Archive/GenABEL/) for genotyped SNPs and ProbABEL^78^ (https://github.com/GenABEL-Project/ProbABEL) for imputed data. SNPs with imputation quality (Rsq in MaCH) < 0.8 or MAF < 0.01 were excluded.

To select linkage disequilibrium (LD)-based independent association signals among the PlGF-associated SNPs from the discovery phase, we conducted the clumping procedure implemented in PLINK^79^ (https://zzz.bwh.harvard.edu/plink/) and picked the index SNPs with the most significant association *p*-value from each clumped association region based on the GWAS. The 1000 Genomes Phase 1 v3 genotypes were used as reference panel; the following thresholds for clumping were applied: association *p*-value < 1 × 10^–6^, physical distance > 1 Mb, and *r*^2^ < 0.01.

Independently associated SNPs in the discovery were *de-novo* genotyped in the 468 individuals of the replication sample using TaqMan SNP genotyping assays, following the manufacturer’s instructions (Bio-Rad, USA).

To assess evidence for replication, test-statistics of discovery and in silico replication samples were meta-analysed using a fixed effect model weighted by inverse variance, using Metal^80^ (http://csg.sph.umich.edu/abecasis/metal/index.html). SNPs were considered replicated if the effect was in the same direction between discovery and replication, and the *p*-value in the meta-analysis was lower than in the discovery sample.

The proportion of phenotypic variance explained by the PlGF-associated variants was estimated fitting 3 linear mixed effect models, in which PlGF levels were regressed, respectively, on: (1) no covariate; (2) gender and age; (3) gender, age and additive effect of each of the three SNPs. The variance explained by the associated variants was estimated using the gaston R package^77,81^ (https://CRAN.R-project.org/package=gaston) *lmm.aireml* function^82^ (https://search.r-project.org/CRAN/refmans/gaston/html/lmm.aireml.html), which uses the genomic kinship matrix to correct for relatedness between individuals.

### Colocalization analysis

Colocalization analysis is a method used to identify shared regulatory variants between a GWAS and an eQTL analysis: if a GWAS trait and a gene expression analysis share the same associated SNP, it may suggest a regulatory role of the SNP mediated through the gene on the GWAS trait.

Colocalization analysis of the PlGF-associated loci with gene expression was conducted using the discovery GWAS results and the cis-eQTL results from 48 tissues in the GTEx Project (Version 7)^20^. We considered the three PlGF-associated loci on chromosomes 7, 10 and 13, and, for each of them, we identified all transcripts and all tissue transcript pairs with reported eQTLs within ± 500 kb of each GWAS index SNP. We used the colocalization method outlined by Giambartolomei et al.^21^, and implemented in the *coloc.abf* function from the R package^77^ *coloc* (https://rdrr.io/cran/coloc/man/coloc.abf.html), applying the default parameters. Evidence for colocalization was defined as an H4 ≥ 0.8, which represents the posterior probability that the association with PlGF and gene expression is due to the same underlying variant.

### Analysis of the imputed genetically regulated gene expression (TWAS)

TWAS is a way of integrating expression data and genome-wide association studies, allowing the discovery of genes associated with traits of interest. TWAS analysis typically consists of two steps: first, a model is trained to predict gene expressions from local genetic variants near the focal genes, using a reference dataset containing both genotype and expression data; then, the pre-trained model is used to predict expressions from genotypes in the dataset under study, which contains genotypes and phenotypes. The predicted expressions are then associated with the phenotype of interest.

The genetically regulated gene expression was imputed using PrediXcan v.7 data^36^ (https://github.com/hakyimlab/PrediXcan). PrediXcan was used to impute the transcriptome of the 1600 individuals who were included in the discovery GWAS using the SNP weights derived from models trained on reference transcriptome datasets of the 48 tissues in the GTEx Project (Version 7)^20^, downloaded from PredictDB, and the genome-wide imputed variants showing MAF > 0.01 and RSQ > 0.80. The residuals of the regression of PlGF levels on sex, age and kinship matrix were used as phenotype. We tested for association 1866–8753 protein-coding genes (depending on the tissue) using a linear regression. An FDR < 0.05 was considered as threshold for statistical significance of associations.

### Gene-based analysis

A gene-based association analysis was performed using VEGAS2 software^22^ (https://vegas2.qimrberghofer.edu.au/). VEGAS2 is an extension of the VErsatile Gene-based Association Study (VEGAS) approach^83^ that uses 1000 Genomes reference data to estimate LD between variants. All the variants from the discovery GWAS were used for the analysis. A ‘ − 20 kbloc’ parameter, which assigns all variants in the gene or within 20 kb on either side of a gene transcription site, was considered. All the variants assigned to a gene were used to compute the gene-based *p*-value. The 1000 Genomes European population was considered for the estimation of LD between variants. In total, 24,009 autosomal genes were analysed.

### Pathway analysis

A pathway enrichment analysis was performed using the GSA-SNP2 software^23^ (https://sourceforge.net/projects/gsasnp2), considering the VEGAS2 gene-based association data as input for the analysis. 6425 Gene Ontology (GO) gene sets, with a pathway size between 10 and 200, were used as reference pathways. To reduce false positives, highly correlated adjacent genes (inter-gene correlations > 0.5) were removed using the European 1000 Genome genotype data as reference. Pathways were considered significantly enriched if they showed a q-value < 0.05. To summarize and reduce functional redundancies in the enrichment results, the REVIGO software^24^ (http://revigo.irb.hr/) was used. The 27 significantly enriched pathways were used as input. A semantic similarity threshold of 0.5 was considered to select non-redundant GO terms.

## Supplementary Information

Supplementary Information.

## References

[1] Carmeliet P (2001). Synergism between vascular endothelial growth factor and placental growth factor contributes to angiogenesis and plasma extravasation in pathological conditions. Nat. Med..

[2] Fischer C, Mazzone M, Jonckx B, Carmeliet P (2008). FLT1 and its ligands VEGFB and PlGF: Drug targets for anti-angiogenic therapy?. Nat. Rev. Cancer.

[3] Van de Veire S (2010). Further pharmacological and genetic evidence for the efficacy of PlGF inhibition in cancer and eye disease. Cell.

[4] Naik A (2017). Neuropilin-1 associated molecules in the blood distinguish poor prognosis breast cancer: A cross-sectional study. Sci. Rep..

[5] Pagani E (2016). Placenta growth factor and neuropilin-1 collaborate in promoting melanoma aggressiveness. Int. J. Oncol..

[6] Schmidt T (2011). Loss or inhibition of stromal-derived PlGF prolongs survival of mice with imatinib-resistant Bcr-Abl1(+) leukemia. Cancer Cell.

[7] Yoo SA (2009). Role of placenta growth factor and its receptor flt-1 in rheumatoid inflammation: A link between angiogenesis and inflammation. Arthritis Rheum..

[8] Zhou L, Lu G, Shen L, Wang L, Wang M (2014). Serum levels of three angiogenic factors in systemic lupus erythematosus and their clinical significance. Biomed Res. Int..

[9] Siervo M (2010). Angiogenesis and biomarkers of cardiovascular risk in adults with metabolic syndrome: Original Article. J. Int. Med..

[10] Fong SW (2015). Systemic and coronary levels of CRP, MPO, sCD40L and PlGF in patients with coronary artery disease. BMC Res. Notes.

[11] Ioanna Z, Christian S, Christian G, Daniel B (2018). Plasma levels of hypoxia-regulated factors in patients with age-related macular degeneration. Graefe’s Arch. Clin. Exp. Ophthalmol..

[12] Matsui M (2015). Placental growth factor as a predictor of cardiovascular events in patients with CKD from the NARA-CKD study. J. Am. Soc. Nephrol..

[13] Wagner, L. K. Diagnosis and management of preeclampsia. *American Family Physician* (2004). 15617295

[14] Maynard SE (2003). Excess placental soluble fms-like tyrosine kinase 1 (sFlt1) may contribute to endothelial dysfunction hypertension, and proteinuria in preeclampsia. J. Clin. Invest..

[15] Levine RJ (2006). Soluble endoglin and other circulating antiangiogenic factors in preeclampsia. N. Engl. J. Med..

[16] Chau K, Hennessy A, Makris A (2017). Placental growth factor and pre-eclampsia. J. Hum. Hypertens..

[17] Sorice R (2012). Genetic and environmental factors influencing the placental growth factor (PGF) variation in two populations. PLoS ONE.

[18] Colonna V (2007). Campora: A young genetic isolate in South Italy. Hum. Hered..

[19] Colonna V (2009). Comparing population structure as inferred from genealogical versus genetic information. Eur. J. Hum. Genet..

[20] Lonsdale J (2013). The genotype-tissue expression (GTEx) project. Nat. Genet..

[21] Giambartolomei C (2014). Bayesian test for colocalisation between pairs of genetic association studies using summary statistics. PLoS Genet..

[22] Mishra A, Macgregor S (2015). VEGAS2: Software for more flexible gene-based testing. Twin Res. Hum. Genet..

[23] Yoon S (2018). Efficient pathway enrichment and network analysis of GWAS summary data using GSA-SNP2. Nucleic Acids Res..

[24] Supek F, Bošnjak M, Škunca N, Šmuc T (2011). Revigo summarizes and visualizes long lists of gene ontology terms. PLoS ONE.

[25] Snuderl M (2013). Targeting placental growth factor/neuropilin 1 pathway inhibits growth and spread of medulloblastoma. Cell.

[26] Tjwa M, Luttun A, Autiero M, Carmeliet P (2003). VEGF and PIGF: Two pleiotropic growth factors with distinct roles in development and homeostasis. Cell Tissue Res..

[27] De Rooij J (2000). Mechanism of regulation of the Epac family of cAMP-dependent RapGEFs. J. Biol. Chem..

[28] Rebhun JF, Castro AF, Quilliam LA (2000). Identification of guanine nucleotide exchange factors (GEFs) for the Rap1 GTPase. J. Biol. Chem..

[29] Boettner B, Van Aelst L (2009). Control of cell adhesion dynamics by Rap1 signaling. Curr. Opin. Cell Biol..

[30] Caron E (2003). Cellular functions of the Rap1 GTP-binding protein: A pattern emerges. J. Cell Sci..

[31] Chrzanowska-Wodnicka M (2010). Regulation of angiogenesis by a small GTPase Rap1. Vascul. Pharmacol..

[32] Tawa H (2010). Role of afadin in vascular endothelial growth factor-and sphingosine 1-phosphate-induced angiogenesis. Circ. Res..

[33] Lakshmikanthan S (2011). Rap1 promotes VEGFR2 activation and angiogenesis by a mechanism involving integrin αvβ3. Blood.

[34] Lakshmikanthan S (2018). Rap1B promotes VEGF-induced endothelial permeability and is required for dynamic regulation of the endothelial barrier. J. Cell Sci..

[35] Vodolazkaia A (2016). Vascular endothelial growth factor pathway in endometriosis: Genetic variants and plasma biomarkers. Fertil. Steril..

[36] Gamazon ER (2015). A gene-based association method for mapping traits using reference transcriptome data. Nat. Genet..

[37] Kong HK, Park JH (2012). Characterization and function of human Ly-6/uPAR molecules. BMB Rep..

[38] Loughner CL (2016). Organization, evolution and functions of the human and mouse Ly6/uPAR family genes. Hum. Genomics.

[39] Upadhyay G (2019). Emerging role of lymphocyte antigen-6 family of genes in cancer and immune cells. Front. Immunol..

[40] Carroll MC (1998). The role of complement and complement receptors in induction and regulation of immunity. Annu. Rev. Immunol..

[41] Ross SC, Densen P (1984). Complement deficiency states and infection: Epidemiology, pathogenesis and consequences of neisserial and other infections in an immune deficiency. Med. (United States).

[42] Lintner KE (2016). Gene copy-number variations (CNVs) of complement C4 and C4A deficiency in genetic risk and pathogenesis of juvenile dermatomyositis. Ann. Rheum. Dis..

[43] Li N (2017). Association between C4, C4A, and C4B copy number variations and susceptibility to autoimmune diseases: A meta-analysis. Sci. Rep..

[44] Mason MJ (2014). Low HERV-K(C4) copy number is associated with type 1 diabetes. Diabetes.

[45] Zeisler H (2016). Predictive value of the sFlt-1:PlGF ratio in women with suspected preeclampsia. N. Engl. J. Med..

[46] Verlohren S (2014). New gestational phase-specific cutoff values for the use of the soluble fms-like tyrosine kinase-1/placental growth factor ratio as a diagnostic test for preeclampsia. Hypertension.

[47] Inkeri Lokki A (2014). Complement activation and regulation in preeclamptic placenta. Front. Immunol..

[48] Lechner J (2016). Higher plasma levels of complement C3a, C4a and C5a increase the risk of subretinal fibrosis in neovascular age-related macular degeneration. Immun. Ageing.

[49] Omori T (2019). Evidence for activation of lectin and classical pathway complement components in aqueous humor of neovascular age-related macular degeneration. Ophthalmic Res..

[50] Astle WJ (2016). The allelic landscape of human blood cell trait variation and links to common complex disease. Cell.

[51] Jin Y (2016). Genome-wide association studies of autoimmune vitiligo identify 23 new risk loci and highlight key pathways and regulatory variants. Nat. Genet..

[52] Jin Y (2010). Variant of TYR and autoimmunity susceptibility loci in generalized vitiligo. N. Engl. J. Med..

[53] Nakabayashi K (2011). Identification of independent risk loci for Graves disease within the MHC in the Japanese population. J. Hum. Genet..

[54] Aterido A (2019). Genetic variation at the glycosaminoglycan metabolism pathway contributes to the risk of psoriatic arthritis but not psoriasis. Ann. Rheum. Dis..

[55] Lu CW, Xiong Y, He P (2007). Dimethylarginine dimethylaminohydrolase-2 overexpression improves impaired nitric oxide synthesis of endothelial cells induced by glycated protein. Nitric Oxide - Biol. Chem..

[56] Fiedler LR, Wojciak-Stothard B (2009). The DDAH/ADMA pathway in the control of endothelial cell migration and angiogenesis. Biochem. Soc. Trans..

[57] Hasegawa K (2006). Dimethylarginine dimethylaminohydrolase 2 increases vascular endothelial growth factor expression through Sp1 transcription factor in endothelial cells. Arterioscler. Thromb. Vasc. Biol..

[58] Niu PP (2014). Hypermethylation of DDAH2 promoter contributes to the dysfunction of endothelial progenitor cells in coronary artery disease patients. J. Transl. Med..

[59] Lange C (2016). Dimethylarginine dimethylaminohydrolase-2 deficiency promotes vascular regeneration and attenuates pathological angiogenesis. Exp. Eye Res..

[60] Anderssohn M (2012). Severely decreased activity of placental dimethylarginine dimethylaminohydrolase in pre-eclampsia. Eur. J. Obstet. Gynecol. Reprod. Biol..

[61] Azizi F (2019). Altered methylation and expression patterns of genes regulating placental nitric oxide pathway in patients with severe preeclampsia. Hum. Antibodies.

[62] Krautkramer KA (2013). Tcf19 is a novel islet factor necessary for proliferation and survival in the INS-1 β-cell line. Am. J. Physiol. Endocrinol. Metab..

[63] Kim YJ (2013). A genome-wide association study identified new variants associated with the risk of chronic hepatitis B. Hum. Mol. Genet..

[64] Cheung YH, Watkinson J, Anastassiou D (2011). Conditional meta-analysis stratifying on detailed HLA genotypes identifies a novel type 1 diabetes locus around TCF19 in the MHC. Hum. Genet..

[65] Mahajan A (2018). Refining the accuracy of validated target identification through coding variant fine-mapping in type 2 diabetes article. Nat. Genet..

[66] Xian Zeng C (2019). TCF19 enhances cell proliferation in hepatocellular carcinoma by activating the ATK/FOXO1 signaling pathway. Neoplasma.

[67] Zhou ZH (2019). TCF19 contributes to cell proliferation of non-small cell lung cancer by inhibiting FOXO1. Cell Biol. Int..

[68] Lin Y-L, Liang Y-C, Chiang B-L (2007). Placental growth factor down-regulates type 1 T helper immune response by modulating the function of dendritic cells. J. Leukoc. Biol..

[69] Han S (2014). Glioma cell-derived placental growth factor induces regulatory B cells. Int. J. Biochem. Cell Biol..

[70] Yoo SA (2019). Placental growth factor regulates the generation of TH17 cells to link angiogenesis with autoimmunity. Nat. Immunol..

[71] Dong C (2008). TH17 cells in development: An updated view of their molecular identity and genetic programming. Nat. Rev. Immunol..

[72] Volin V, Shahrara S (2011). Role of TH-17 cells in rheumatic and other autoimmune diseases. Rheumatol. Curr. Res..

[73] Kang M (2018). Placental growth factor (PlGF) is linked to inflammation and metabolic disorders in mice with diet-induced obesity. Endocr. J..

[74] Li Y, Willer CJ, Ding J, Scheet P, Abecasis GR (2010). MaCH: Using sequence and genotype data to estimate haplotypes and unobserved genotypes. Genet. Epidemiol..

[75] Howie B, Fuchsberger C, Stephens M, Marchini J, Abecasis GR (2012). Fast and accurate genotype imputation in genome-wide association studies through pre-phasing. Nat. Genet..

[76] Aulchenko YS, Ripke S, Isaacs A, van Duijn CM (2007). GenABEL: An R library for genome-wide association analysis. Bioinformatics.

[77] R Development Core Team. R: A language and environment for statistical computing. *R Found. Stat. Comput. Vienna, Austria.* ISBN 3-900, (2017).

[78] Aulchenko YS, Struchalin MV, van Duijn CM (2010). ProbABEL package for genome-wide association analysis of imputed data. BMC Bioinform..

[79] Purcell S (2007). PLINK: A tool set for whole-genome association and population-based linkage analyses. Am. J. Hum. Genet..

[80] Willer CJ, Li Y, Abecasis GR (2010). METAL: Fast and efficient meta-analysis of genomewide association scans. Bioinformatics.

[81] 46th European Mathematical Genetics Meeting (EMGM) 2018, Cagliari, Italy, April 18–20, 2018: Abstracts. *Hum. Hered.* (2018) 10.1159/000488519. 10.1159/000488519 29669356

[82] Gilmour, A. R., Thompson, R. & Cullis, B. R. Average information REML: An efficient algorithm for variance parameter estimation in linear mixed models. *Biometrics* **51**, (1995).

[83] Liu JZ (2010). A versatile gene-based test for genome-wide association studies. Am. J. Hum. Genet..

