# Supplementary material for: Genetics of PlGF plasma levels highlights a role of its receptors and supports the link between angiogenesis and immunity
Source: Sci Rep. 2021 Aug 19;11:16821. doi: 10.1038/s41598-021-96256-0 (PMC8376970; doi:10.1038/s41598-021-96256-0)
Supplement: Supplementary file 1 — Supplementary Information. [file 41598_2021_96256_MOESM1_ESM.docx]

**Supporting information**

**for**

**Genetics of PlGF plasma levels highlights a role of its receptors and supports the link between angiogenesis and immunity**

Daniela Ruggiero^1,2^*, Teresa Nutile^1^, Stefania Nappo^3^, Alfonsina Tirozzi^2^, Celine Bellenguez^4^, Anne-Louise Leutenegger^5,6^, Marina Ciullo^1,2^*

*1. Institute of Genetics and Biophysics, National Research Council of Italy, Naples, Italy*

*2. IRCCS Neuromed, Pozzilli, Isernia, Italy*

*3. AORN Santobono-Pausilipon Hospital, Naples, Italy*

*4. Inserm, Institut Pasteur de Lille, Univ. Lille, CHU Lille, U1167 - Labex DISTALZ - RID-AGE - Risk factors and molecular determinants of aging-related diseases, F-59000 Lille, France*

*5. Inserm, UMR 946, Genetic variation and Human diseases, F-75010, Paris, France*

*6.* *Université Paris-Diderot, Sorbonne Paris Cité, UMR946, F-75010, Paris, France*

**
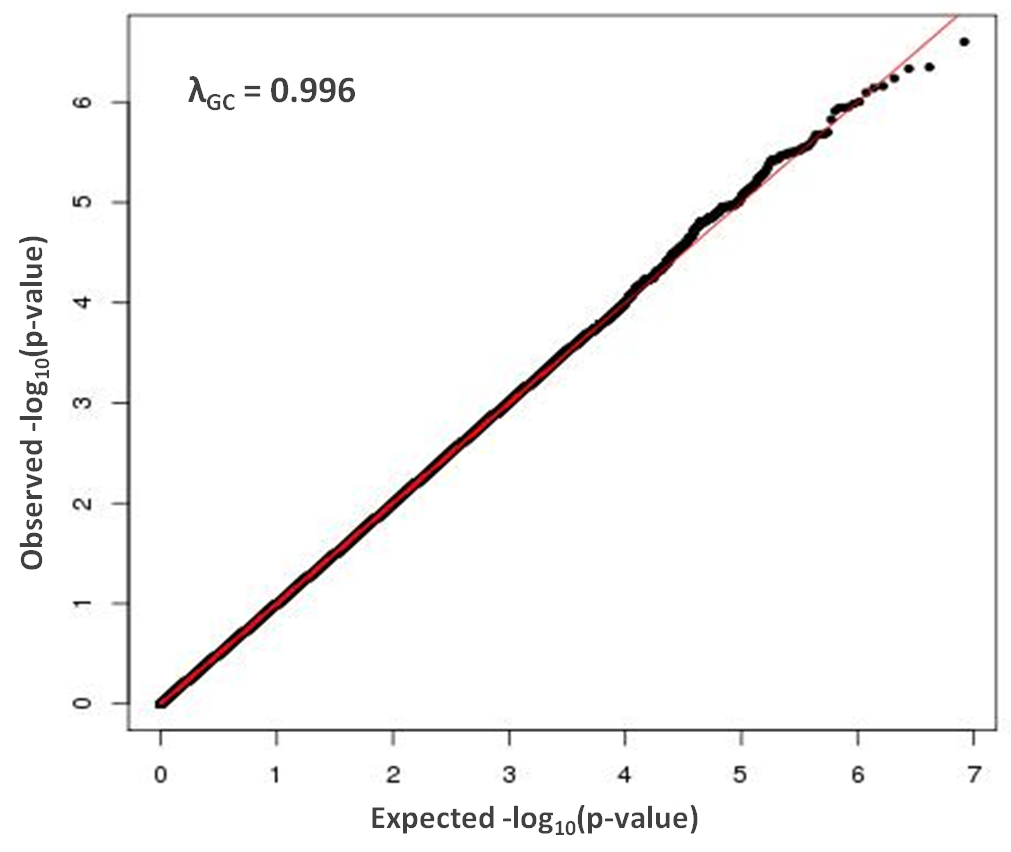
**

**S1 Figure**. Quantile-Quantile plot for the PlGF levels GWAS in the Discovery sample. λ_GC_ is the genomic inflation factor for the discovery GWAS p-values.


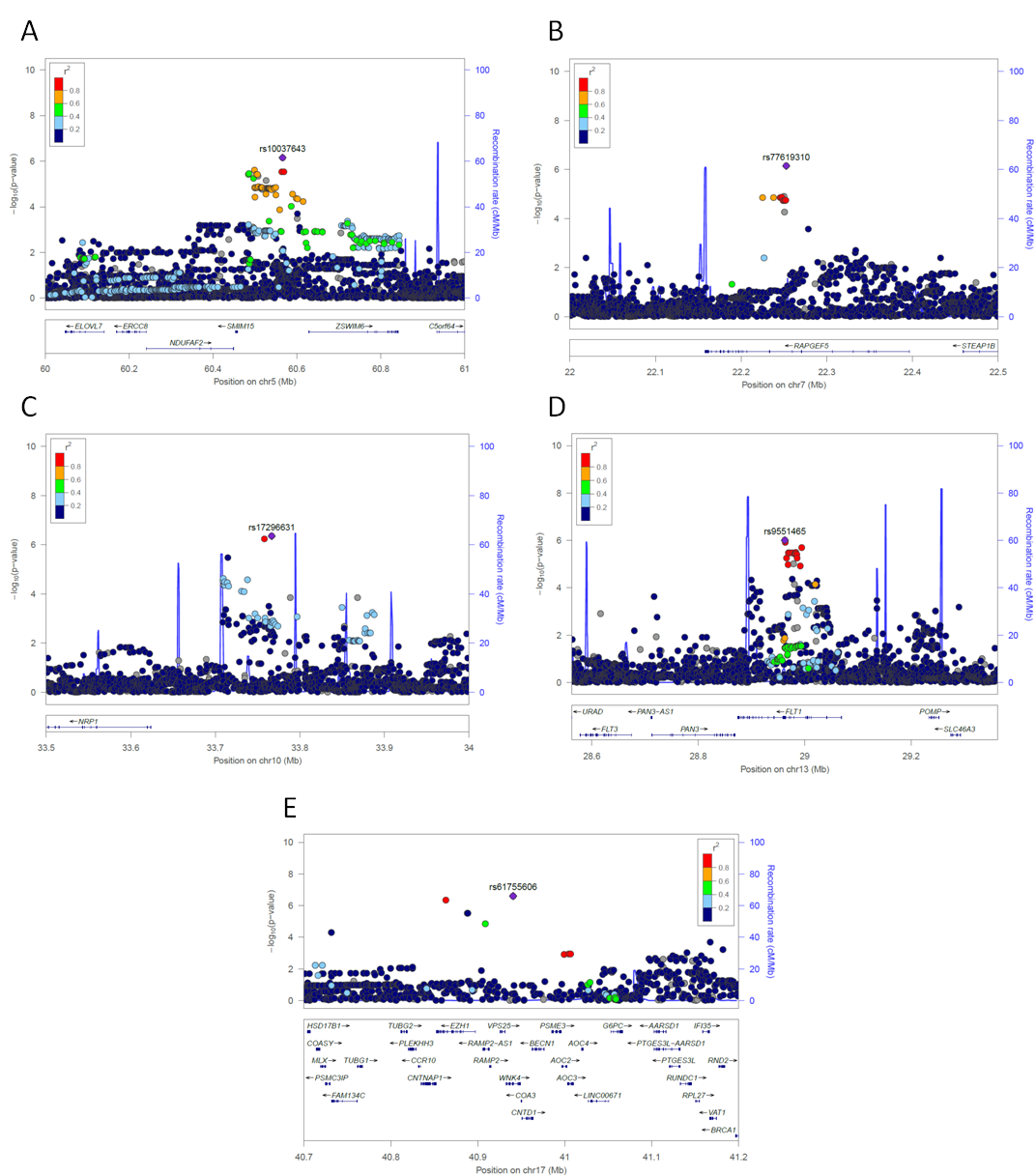


**S2 Figure**. Regional plots of top 5 variants in the Discovery GWAS. Regional association plots show –log10 p-values for all variants ordered by their chromosomal position (GRCh37) within regions of the 5 associated variants. Panels A-E show the 5q12.1, 7p15.3, 10p11.22, 13q12.3 and 17q21.31 loci, respectively. The variants were color coded by R-square values with the top variant shaped in diamond. The estimated recombination rates (cM/Mb) were generated from HapMap Phase II genetic map and showed in blue line. The bottom panel of each plot shows the name and location of genes in the UCSC Genome Browser.

**S1 Table.** Significantly associated genes from TWAS analysis.

| **Tissue** | **Gene** | **Chr** | **Gene Start (hg19)** | **Genomic Band** | **Beta** | **SE** | **z** | **p-value** | **FDR** |
| --- | --- | --- | --- | --- | --- | --- | --- | --- | --- |
| Esophagus Gastroesophageal Junction | *PLEKHA6* | 1 | 204187979 | q32.1 | -0.079 | 0.018 | -4.441 | 9.56x10^-6^ | 0.048 |
| Small Intestine Terminal Ileum | *SCGB3A1* | 5 | 180017103 | q35.3 | 0.153 | 0.033 | 4.672 | 3.23x10^-6^ | 0.046 |
| Muscle Skeletal | *HLA-K* | 6 | 29894236 | p22.1 | 0.082 | 0.017 | 4.892 | 1.10x10^-6^ | 0.039 |
| Artery Coronary | *HLA-K* | 6 | 29894236 | p22.1 | 0.123 | 0.027 | 4.630 | 3.95x10^-6^ | 0.046 |
| Artery Coronary | *MICD* | 6 | 29938149 | p22.1 | 0.157 | 0.033 | 4.785 | 1.87x10^-6^ | 0.041 |
| Esophagus Gastroesophageal Junction | *TCF19* | 6 | 31126303 | p21.33 | 0.128 | 0.024 | 5.290 | 1.40x10^-7^ | 0.026 |
| Adipose Subcutaneous | *TCF19* | 6 | 31126303 | p21.33 | 0.112 | 0.024 | 4.596 | 4.64x10^-6^ | 0.047 |
| Colon Transverse | *TCF19* | 6 | 31126303 | p21.33 | 0.122 | 0.027 | 4.490 | 7.63x10^-6^ | 0.048 |
| Adipose Visceral Omentum | *LY6G5B* | 6 | 31637944 | p21.33 | -0.069 | 0.013 | -5.139 | 3.11x10^-7^ | 0.026 |
| Artery Tibial | *LY6G5B* | 6 | 31637944 | p21.33 | -0.055 | 0.012 | -4.604 | 4.47x10^-6^ | 0.047 |
| Brain Cortex | *LY6G5B* | 6 | 31637944 | p21.33 | -0.125 | 0.028 | -4.461 | 8.73x10^-6^ | 0.048 |
| Muscle Skeletal | *LY6G5B* | 6 | 31637944 | p21.33 | -0.044 | 0.010 | -4.538 | 6.09x10^-6^ | 0.048 |
| Pituitary | *LY6G5B* | 6 | 31637944 | p21.33 | -0.118 | 0.026 | -4.532 | 6.28x10^-6^ | 0.048 |
| Spleen | *LY6G5B* | 6 | 31637944 | p21.33 | -0.229 | 0.051 | -4.491 | 7.61x10^-6^ | 0.048 |
| Adipose Subcutaneous | *LY6G5C* | 6 | 31644461 | p21.33 | -0.055 | 0.012 | -4.434 | 9.90x10^-6^ | 0.048 |
| Artery Aorta | *LY6G5C* | 6 | 31644461 | p21.33 | -0.152 | 0.032 | -4.791 | 1.82x10^-6^ | 0.041 |
| Brain Anterior cingulate cortex BA24 | *LY6G5C* | 6 | 31644461 | p21.33 | -0.032 | 0.007 | -4.454 | 9.03x10^-6^ | 0.048 |
| Brain Caudate basal ganglia | *LY6G5C* | 6 | 31644461 | p21.33 | -0.048 | 0.011 | -4.497 | 7.39x10^-6^ | 0.048 |
| Brain Cerebellar Hemisphere | *LY6G5C* | 6 | 31644461 | p21.33 | -0.030 | 0.006 | -4.996 | 6.49x10^-7^ | 0.032 |
| Brain Cortex | *LY6G5C* | 6 | 31644461 | p21.33 | -0.050 | 0.011 | -4.515 | 6.80x10^-6^ | 0.048 |
| Brain Frontal Cortex BA9 | *LY6G5C* | 6 | 31644461 | p21.33 | -0.063 | 0.014 | -4.481 | 7.95x10^-6^ | 0.048 |
| Brain Hippocampus | *LY6G5C* | 6 | 31644461 | p21.33 | -0.095 | 0.021 | -4.478 | 8.08x10^-6^ | 0.048 |
| Brain Hypothalamus | *LY6G5C* | 6 | 31644461 | p21.33 | -0.047 | 0.011 | -4.482 | 7.91x10^-6^ | 0.048 |
| Brain Nucleus accumbens basal ganglia | *LY6G5C* | 6 | 31644461 | p21.33 | -0.036 | 0.008 | -4.592 | 4.74x10^-6^ | 0.047 |
| Brain Putamen basal ganglia | *LY6G5C* | 6 | 31644461 | p21.33 | -0.036 | 0.008 | -4.642 | 3.73x10^-6^ | 0.046 |
| Brain Substantia nigra | *LY6G5C* | 6 | 31644461 | p21.33 | -0.117 | 0.026 | -4.532 | 6.29x10^-6^ | 0.048 |
| Breast Mammary Tissue | *LY6G5C* | 6 | 31644461 | p21.33 | -0.058 | 0.012 | -4.734 | 2.39x10^-6^ | 0.043 |
| Colon Sigmoid | *LY6G5C* | 6 | 31644461 | p21.33 | -0.107 | 0.022 | -4.926 | 9.27x10^-7^ | 0.038 |
| Esophagus Gastroesophageal Junction | *LY6G5C* | 6 | 31644461 | p21.33 | -0.169 | 0.038 | -4.452 | 9.08x10^-6^ | 0.048 |
| Pituitary | *LY6G5C* | 6 | 31644461 | p21.33 | -0.077 | 0.017 | -4.471 | 8.34x10^-6^ | 0.048 |
| Prostate | *LY6G5C* | 6 | 31644461 | p21.33 | -0.069 | 0.014 | -4.742 | 2.30x10^-6^ | 0.043 |
| Skin Sun Exposed Lower leg | *LY6G5C* | 6 | 31644461 | p21.33 | -0.050 | 0.011 | -4.479 | 8.04x10^-6^ | 0.048 |
| Testis | *LY6G5C* | 6 | 31644461 | p21.33 | -0.140 | 0.028 | -5.067 | 4.50x10^-7^ | 0.028 |
| Thyroid | *LY6G5C* | 6 | 31644461 | p21.33 | -0.060 | 0.013 | -4.625 | 4.05x10^-6^ | 0.046 |
| Whole Blood | *LY6G5C* | 6 | 31644461 | p21.33 | -0.044 | 0.010 | -4.551 | 5.74x10^-6^ | 0.048 |
| Brain Cerebellum | *DDAH2* | 6 | 31694815 | p21.33 | -0.084 | 0.018 | -4.661 | 3.40x10^-6^ | 0.046 |
| Heart Atrial Appendage | *DDAH2* | 6 | 31694815 | p21.33 | -0.145 | 0.032 | -4.526 | 6.46x10^-6^ | 0.048 |
| Artery Tibial | *C4A* | 6 | 31949801 | p21.33 | -0.112 | 0.024 | -4.698 | 2.85x10^-6^ | 0.046 |
| Stomach | *LCN12* | 9 | 139844003 | q34.3 | -0.085 | 0.019 | -4.429 | 1.01x10^-5^ | 0.048 |
| Esophagus Gastroesophageal Junction | *LINC02407* | 12 | 76259839 | q21.2 | 0.190 | 0.039 | 4.857 | 1.31x10^-6^ | 0.041 |
| Artery Tibial | *DAD1* | 14 | 23033805 | q11.2 | -0.226 | 0.051 | -4.429 | 1.01x10^-5^ | 0.048 |
| Skin Sun Exposed Lower leg | *DHRS4* | 14 | 24422795 | q11.2 | -0.072 | 0.016 | -4.436 | 9.78x10^-6^ | 0.048 |
| Skin Not Sun Exposed Suprapubic | *DHRS4L2* | 14 | 24439083 | q11.2 | -0.068 | 0.013 | -5.150 | 2.93x10^-7^ | 0.026 |
| Adipose Subcutaneous | *GPHN* | 14 | 66974125 | q23.3 | 0.119 | 0.025 | 4.775 | 1.96x10^-6^ | 0.041 |
| Heart Left Ventricle | *ATP6V1D* | 14 | 67761088 | q23.3 | 0.851 | 0.178 | 4.783 | 1.89x10^-6^ | 0.041 |
| Muscle Skeletal | *TMEM229B* | 14 | 67913801 | q24.1 | 0.143 | 0.031 | 4.663 | 3.38x10^-6^ | 0.046 |
| Artery Aorta | *PIGH* | 14 | 68048672 | q24.1 | -0.161 | 0.036 | -4.491 | 7.59x10^-6^ | 0.048 |
| Lung | *VTI1B* | 14 | 68113792 | q24.1 | -0.466 | 0.105 | -4.432 | 9.97x10^-6^ | 0.048 |
| Brain Caudate basal ganglia | *CYBA* | 16 | 88709691 | q24.2 | 0.062 | 0.013 | 4.635 | 3.87x10^-6^ | 0.046 |
| Brain Nucleus accumbens basal ganglia | *CYBA* | 16 | 88709691 | q24.2 | 0.069 | 0.015 | 4.565 | 5.39x10^-6^ | 0.048 |
| Whole Blood | *ACOX1* | 17 | 73937588 | q25.1 | 0.066 | 0.015 | 4.457 | 8.90x10^-6^ | 0.048 |
| Artery Tibial | *MISP3* | 19 | 14183821 | p13.12 | -0.09 | 0.020 | -4.566 | 5.36x10^-6^ | 0.048 |

Abbreviations: Chr: Chromosome; SE: Standard Error; z: PrediXcan association z-score; FDR: False Discovery Rate adjusted p-value.

**S2 Table.** Top 10 genes from VEGAS2 gene-based analysis.

| **Chr** | **Gene** | **nSNPs** | **nSims** | **Start** | **Stop** | **p-value** | **FDR** | **Top SNP** | **Top SNP p-value** |
| --- | --- | --- | --- | --- | --- | --- | --- | --- | --- |
| 13 | *FLT1* | 474 | 1.00x10^6^ | 28854482 | 29089265 | 5.10x10^-5^ | 0.66 | rs9551465 | 9.93x10^-7^ |
| 5 | *SMIM15-AS1* | 261 | 1.00x10^6^ | 60438142 | 60547907 | 5.60x10^-5^ | 0.66 | rs171748 | 2.36x10^-6^ |
| 6 | *HLA-DRB5* | 534 | 1.00x10^6^ | 32465153 | 32518006 | 1.84x10^-4^ | 0.68 | rs113739809 | 1.33x10^-5^ |
| 6 | *LY6G5B* | 46 | 1.00x10^6^ | 31618727 | 31660227 | 2.30x10^-4^ | 0.68 | rs116452784 | 4.79x10^-4^ |
| 6 | *ABHD16A* | 64 | 1.00x10^6^ | 31634725 | 31691137 | 2.46x10^-4^ | 0.68 | rs115090578 | 3.14x10^-4^ |
| 6 | *CSNK2B* | 50 | 1.00x10^6^ | 31613656 | 31657847 | 2.52x10^-4^ | 0.68 | rs116452784 | 4.79x10^-4^ |
| 6 | *LY6G6F* | 50 | 1.00x10^6^ | 31654683 | 31698372 | 2.60x10^-4^ | 0.68 | rs115090578 | 3.14x10^-4^ |
| 6 | *LY6G5C* | 52 | 1.00x10^6^ | 31624460 | 31668150 | 2.81x10^-4^ | 0.68 | rs147773310 | 4.75x10^-4^ |
| 6 | *MIR4646* | 47 | 1.00x10^6^ | 31648805 | 31688868 | 2.85x10^-4^ | 0.68 | rs115090578 | 3.14x10^-4^ |
| 6 | *LY6G6E* | 49 | 1.00x10^6^ | 31659752 | 31701842 | 3.04x10^-4^ | 0.68 | rs115090578 | 3.14x10^-4^ |

Abbreviations: Chr: Chromosome; nSNPs: Number of SNPs in the gene; nSims: Number of simulations; FDR: False Discovery Rate adjusted p-value; Start-Stop: gene start and stop positions (hg19) ±20kb; Top SNP: Best associated SNP in each gene.
